# Supplementary material for: TET knockout cells transit between pluripotent states and exhibit precocious germline entry
Source: EMBO J. 2025 Oct 27;44(23):7060–89. doi: 10.1038/s44318-025-00597-9 (PMC12669618; doi:10.1038/s44318-025-00597-9)
Supplement: Supplementary file 4 — Expanded View Figures [file 44318_2025_597_MOESM4_ESM.pdf]

Expanded View Figures

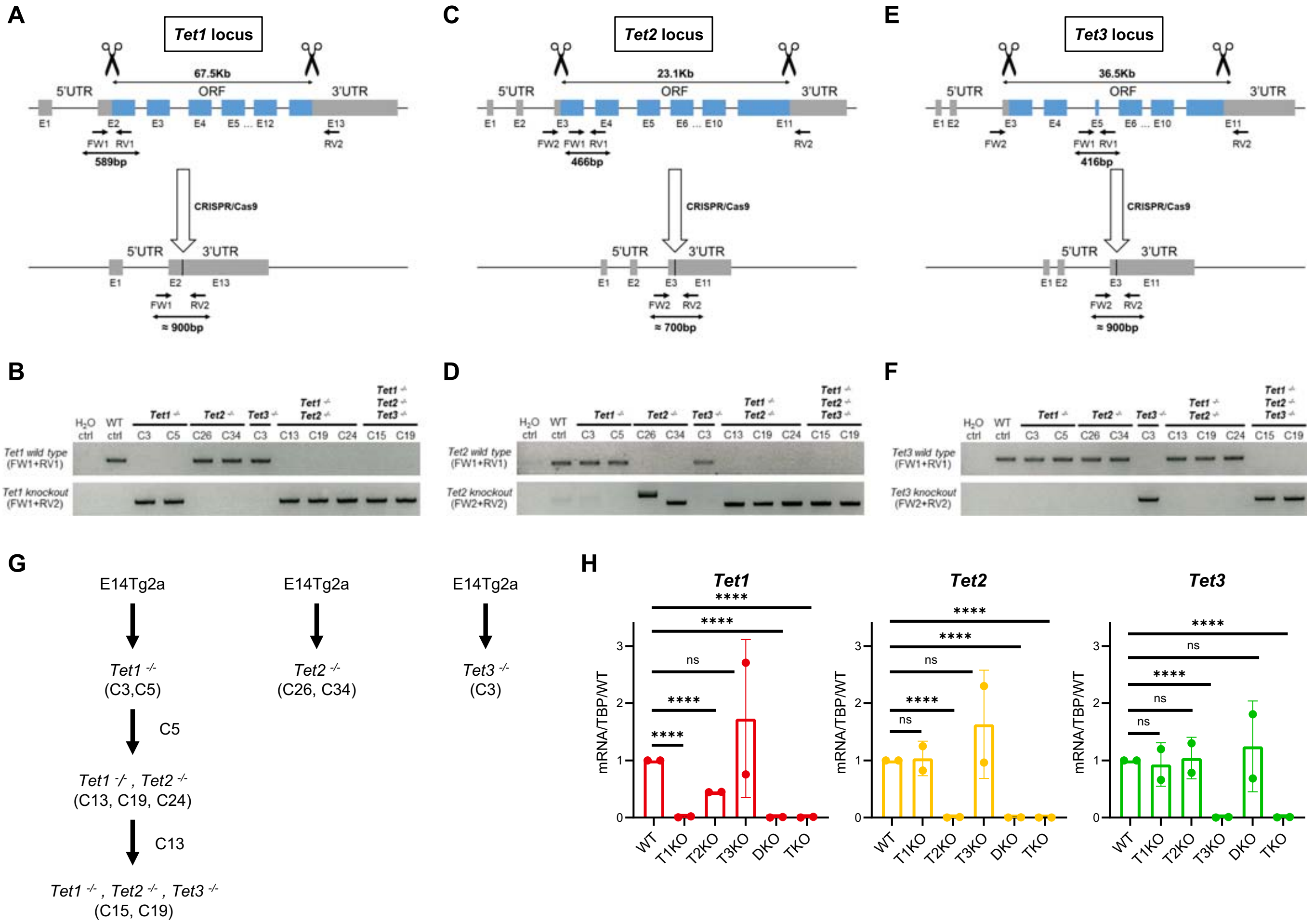

Figure EV1. Genetic deletion of *Tet1/2/3* open reading frames by CRISPR/Cas9.

For all loci the general targeting strategy is indicated (**A**, **C**, **E**) with two gRNAs (scissors) designed close to the start and stop codons, respectively. The position of genotyping primers and the sizes of PCR products are indicated. Open reading frames are indicated in blue and untranslated regions (UTRs) in grey. (**B**, **D**, **F**) Agarose gel analysis of PCR genotyping of *Tet1, 2* and *3* alleles in all cell lines, with the PCR reaction indicated on the left, and the deduced genotype at the top. (**A**) To knockout *Tet1*, wild-type E14Tg2a ESCs were co-transfected with Cas9 and two gRNAs targeting the *Tet1* start and stop codons, respectively. (**B**) Two $Tet1^{-/-}$ clones carrying deletions of both *Tet1* alleles (C3 and C5) were obtained, as demonstrated by the presence of a PCR product for the knockout allele (FW1 + RV2) and the absence of a PCR product for the wild-type allele (FW1 + RV1). (**C**) *Tet2* knockout and *Tet1, Tet2* double knockout (hereafter referred to as DKO) cell lines were generated from E14Tg2a and $Tet1^{-/-}$ C5 ESCs using the strategy shown. (**D**) Clones (C26, C34) lacking *Tet2* alleles and clones lacking both *Tet1* and *Tet2* alleles (C13, C19 and C24) were generated. (**E**) *Tet3* knockout and *Tet1, Tet2* and *Tet*3 triple knockout (hereafter referred to as TKO) ESCs lacking all six *Tet* alleles were generated from E14Tg2a and $Tet1^{-/-}$, $Tet2^{-/-}$ C13 ESCs as illustrated. (**F**) One clone lacking *Tet3* alleles (C3) and two TKO clones lacking all alleles for *Tet1, Tet2* and *Tet3* (C15, C19) were obtained. WT ctrl. Wild-type E14Tg2a ESCs (parental cell line), H_2O ctrl. Control sample with no DNA template. (**G**) Summary of the *Tet* knockout ESC lines generated in this study and their interrelationships (see Fig. 1). Homozygous clone numbers are indicated within brackets. New rounds of targeting are indicated with arrows. (**H**) *Tet1*, *Tet2* and *Tet3* mRNA levels in the indicated ESC lines. mRNA levels were quantified by RT-qPCR, normalised to TBP mRNA levels, and expressed relative to levels in wild-type E14Tg2a ESCs (mRNA/TBP/WT ESC). Data points: replicate experiments, centre: mean, error bars: standard deviation, $n = 2$. Stars indicate statistical significance compared to wild-type (Student's *t*-test). Individual *p* values are provided in Table EV1.

 © The Author(s)

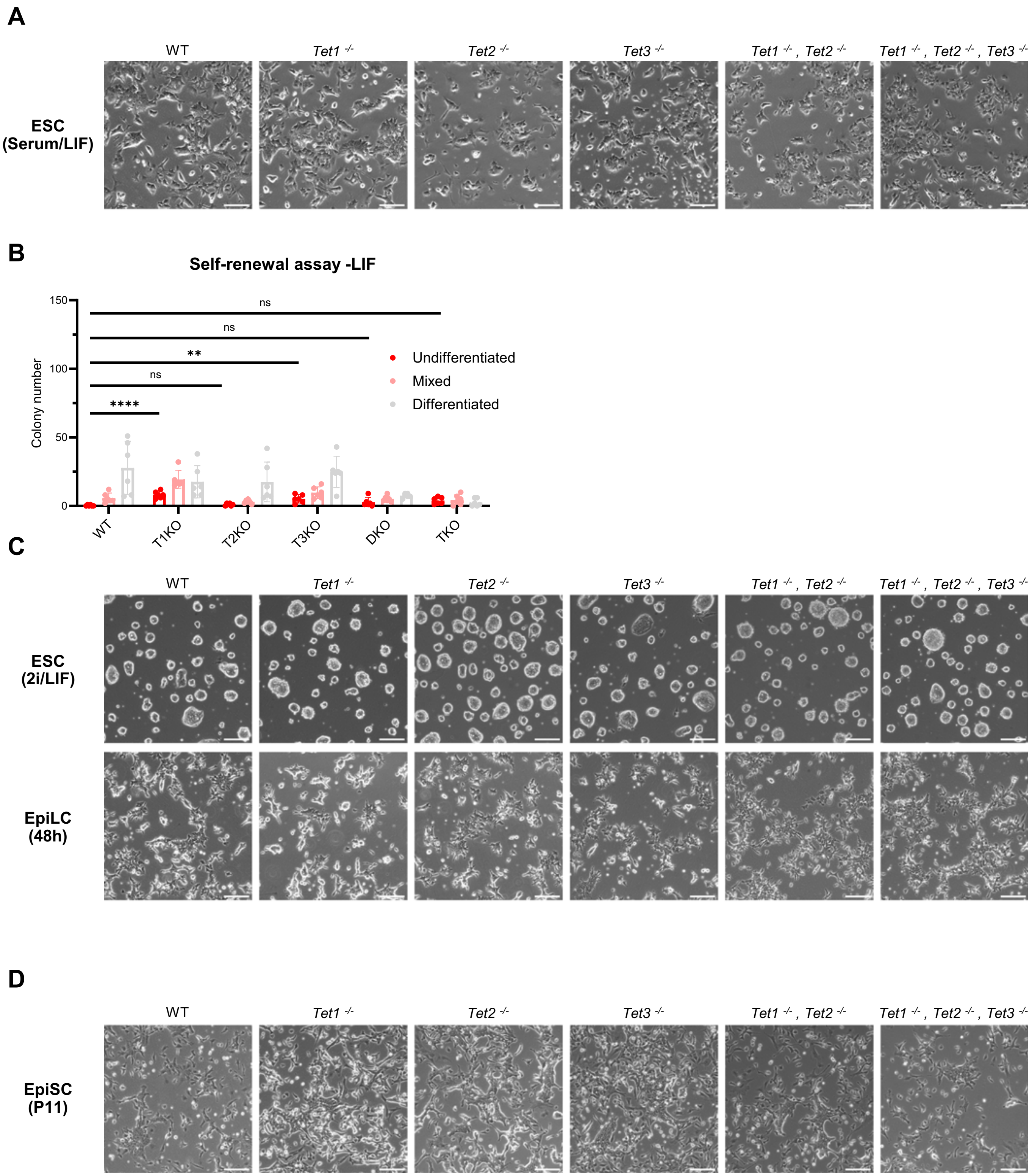

Figure EV2. Phenotype of TET-deficient lines in different pluripotent states.

(**A**) Phase contrast images of the indicated live ESC lines cultured in serum/LIF. Scale bars: 100 μm. (**B**) Alkaline phosphatase staining of the indicated ESC lines following plating at clonal density in serum-containing medium without LIF for 7 days. Colonies were counted and categorised according to their alkaline phosphatase staining (data points: replicate wells, centre: mean, error bars: standard deviation, $n = 6$). Stars indicate statistical significance compared to wild-type for undifferentiated colonies (one-way ANOVA test). Individual p-values are provided in Table EV1. (**C**) Phase contrast images of the indicated live ESC lines cultured in 2i/LIF and following 48 h of EpiLC differentiation in activin/FGF. Scale bars:100 μm. (**D**) Phase contrast images of the indicated live EpiSC lines (passage 11) cultured in activin/FGF. Scale bars:100 μm.

© The Author(s)

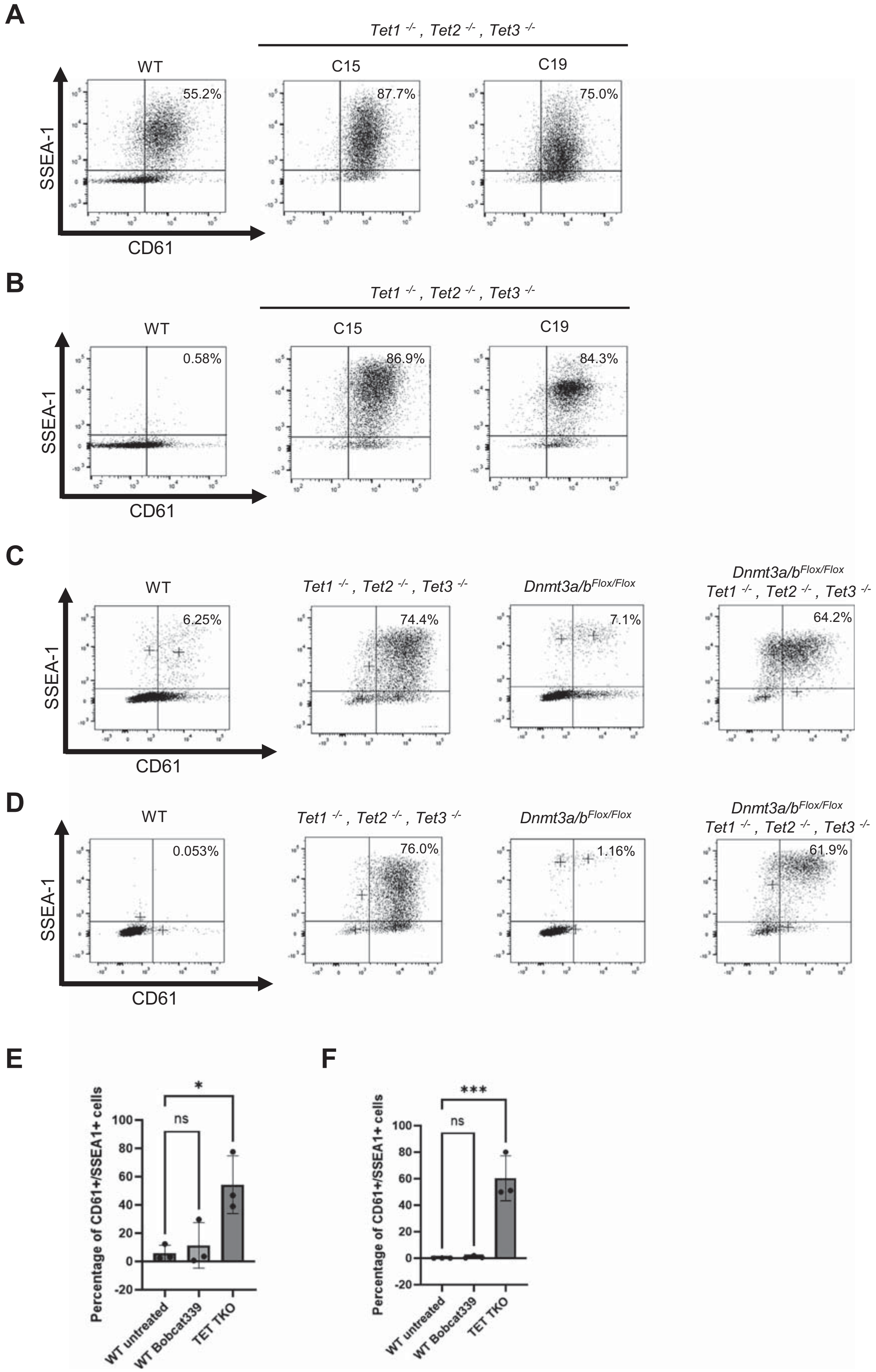
A
Tet1 -/- , Tet2 -/- , Tet3 -/-
WT
C15
C19
55.2%
87.7%
75.0%
SSEA-1
CD61
B
Tet1 -/- , Tet2 -/- , Tet3 -/-
WT
C15
C19
0.58%
86.9%
84.3%
SSEA-1
CD61
C
WT
Tet1 -/- , Tet2 -/- , Tet3 -/-
Dnmt3a/b Flox/Flox
Dnmt3a/b Flox/Flox Tet1 -/- , Tet2 -/- , Tet3 -/-
6.25%
74.4%
7.1%
64.2%
SSEA-1
CD61
D
WT
Tet1 -/- , Tet2 -/- , Tet3 -/-
Dnmt3a/b Flox/Flox
Dnmt3a/b Flox/Flox Tet1 -/- , Tet2 -/- , Tet3 -/-
0.053%
76.0%
1.16%
61.9%
SSEA-1
CD61
E
Percentage of CD61+/SSEA1+ cells
*
ns
WT untreated
WT Bobcat339
TET TKO
F
Percentage of CD61+/SSEA1+ cells
***
ns
WT untreated
WT Bobcat339
TET TKO

 © The Author(s)

Figure EV3. Phenotype of independent TET-TKO lines in germline commitment.

(**A**, **B**) Flow cytometric analysis of independent clones (C15 and C19) of *Tet1*$^{-/-}$, *Tet2*$^{-/-}$, *Tet3*$^{-/-}$ (TKO) cell line following 6 days of PGCLC differentiation with (**A**) or without (**B**) PGC-promoting cytokines. SSEA-1/CD61 double-positive cells in the top right quadrant report PGCLCs. (**C**, **D**) Flow cytometric analysis of indicated cell lines following 6 days of PGCLC differentiation with (**C**) or without (**D**) PGC-promoting cytokines. SSEA-1/CD61 double-positive cells in the top right quadrant report PGCLCs. Cell lines used: WT: E14Tg2a, *Tet1*$^{-/-}$, *Tet2*$^{-/-}$, *Tet3*$^{-/-}$: TET triple knockout line generated in this study, *Dnmt3a/b*$^{Flox/Flox}$: wild-type cell line (Ginno et al, 2020), *Dnmt3a/b*$^{Flox/Flox}$ *Tet1*$^{-/-}$, *Tet2*$^{-/-}$, *Tet3*$^{-/-}$: TET triple knockout line generated by Ginno et al, 2020. (**E**, **F**) Quantification of SSEA-1/CD61 double-positive cells following 6 days of PGCLC differentiation with (**E**) or without (**F**) cytokines in the indicated cell line and treatment (data points: three biological replicates, centre: mean, error bars: standard deviation). Stars indicate statistical significance compared to wild-type (one-way ANOVA test). Individual *p* values are provided in Table EV1.

© The Author(s)

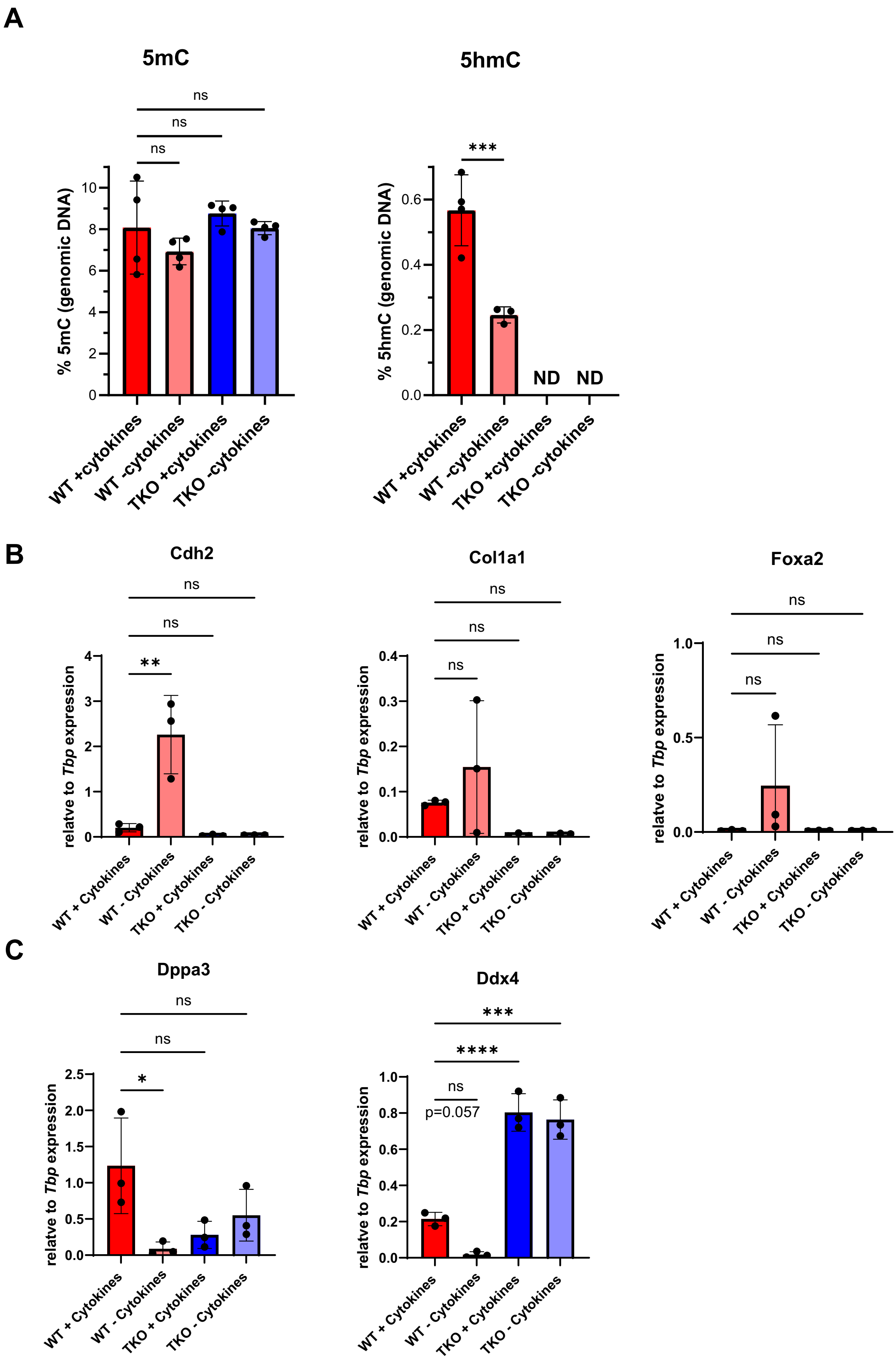
A
5mC
5hmC
ns
ns
ns
***
% 5mC (genomic DNA)
% 5hmC (genomic DNA)
ND
ND
WT +cytokines
WT -cytokines
TKO +cytokines
TKO -cytokines
B
Cdh2
Col1a1
Foxa2
**
relatve to Tbp expression
WT + Cytokines
WT - Cytokines
TKO + Cytokines
TKO - Cytokines
C
Dppa3
Ddx4
*
****
p=0.057

 © The Author(s)

Figure EV4. Further characterisation of TET-TKO compared to wild-type PGCLCs.

(**A**) 5mC and 5hmC levels measured by ELISA in wild-type and TKO cells at day 6 of differentiation (+/− cytokines). Wild-type cells differentiated in the presence of cytokines were sorted into SSEA1^{+}/CD61^{+} populations to select for PGCLCs. All other populations were collected as full aggregates (data points: technical and biological replicates, centre: mean, error bars: standard deviation, $n = 2$). Stars indicate statistical significance compared to wild-type (one-way ANOVA test). Individual p values are provided in Table EV1. (**B**, **C**) mRNA levels of neural marker *Cdh2*, mesodermal marker *Col1a1*, endoderm marker *Foxa2* (**B**) and the early germline marker *Dppa3* and the late marker *Ddx4*. (**C**) RNA isolated from wild-type cells differentiated in the presence of cytokines and TET-TKO cells differentiated either in the presence or absence of cytokines sorted into SSEA1^{+}/CD61^{+} populations to select for PGCLCs. Wild-type cells differentiated in the absence of cytokines were collected as full aggregates. mRNA levels were quantified by RT-qPCR and normalised to TBP mRNA levels (data points: three biological replicates, centre: mean, error bars: standard deviation, $n = 3$). Stars indicate statistical significance compared to wild-type + cytokines (Student's t-test). Individual p values are provided in Table EV1.

© The Author(s)

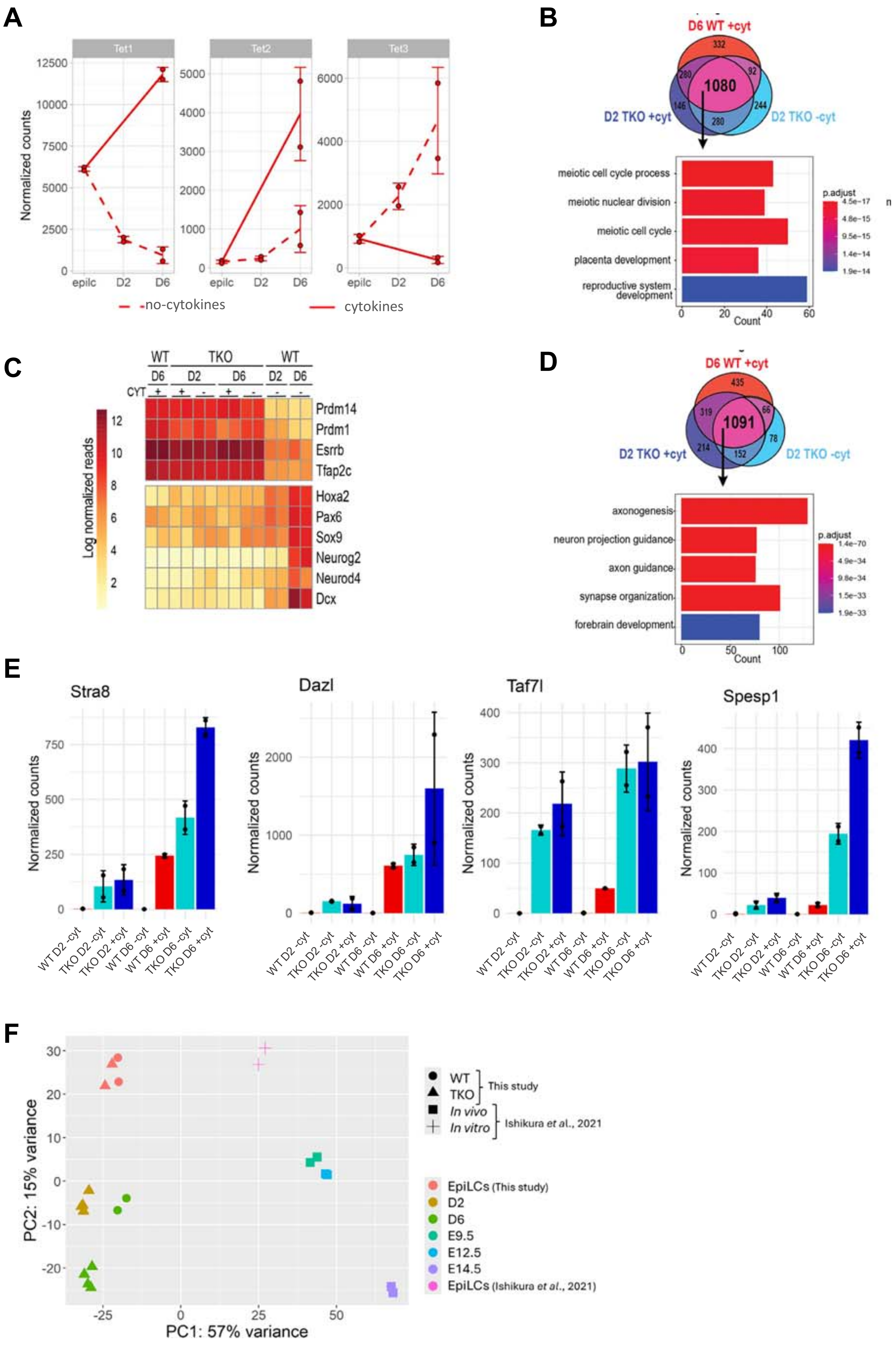
A
Tet1
Tet2
Tet3
Normalized counts
epilc D2 D6
no-cytokines
cytokines
B
D6 WT +cyt
D2 TKO +cyt
D2 TKO -cyt
1080
meiotic cell cycle process
meiotic nuclear division
meiotic cell cycle
placenta development
reproductive system development
Count
p.adjust
C
WT TKO WT
D6 D2 D6 D2 D6
CYT
Log normalized reads
Prdm14
Prdm1
Esrrb
Tfap2c
Hoxa2
Pax6
Sox9
Neurog2
Neurod4
Dcx
D
D6 WT +cyt
D2 TKO +cyt
D2 TKO -cyt
1091
axonogenesis
neuron projection guidance
axon guidance
synapse organization
forebrain development
Count
p.adjust
E
Stra8
Dazl
Taf7l
Spesp1
Normalized counts
WT D2 -cyt
TKO D2 -cyt
TKO D2 +cyt
WT D6 -cyt
WT D6 +cyt
TKO D6 -cyt
TKO D6 +cyt
F
PC2: 15% variance
PC1: 57% variance
WT
TKO
This study
In vivo
In vitro
Ishikura et al., 2021
EpiLCs (This study)
D2
D6
E9.5
E12.5
E14.5
EpiLCs (Ishikura et al., 2021)

 © The Author(s)

Figure EV5. Timecourse RNA-seq analysis during TET triple knockout PGCLC differentiation.

(**A**) Expression dynamics of *Tet1, Tet2* and *Tet3* in wild-type cells during PGCLC differentiation in the presence and absence of cytokines (RNA-seq reads normalised by DESeq2). Data points: biological replicates, line: mean, error bars: standard deviation, $n = 2$. (**B**) Overlap of all genes detected as significantly upregulated in day 6 wild-type PGCLCs and day 2 *Tet1*$^{-/-}$, *Tet2*$^{-/-}$, *Tet3*$^{-/-}$ TKO (+/− cytokines) compared to day 6 wild-type cells differentiated without cytokines (q value <0.05, Log2 fold change >2). Gene ontology enrichment results are shown for genes shared across all three samples. (**C**) Log-normalised reads of germline and somatic markers. (**D**) Overlap of all genes detected as significantly downregulated in day 6 wild-type PGCLCs and day 2 *Tet1*$^{-/-}$, *Tet2*$^{-/-}$, *Tet3*$^{-/-}$ TKO (+/− cytokines) compared to day 6 wild-type cells differentiated without cytokines (q value <0.05, Log2 fold change <−2). Gene ontology enrichment results are shown for genes shared across all three samples.
(**E**) Expression of late germline markers in wild-type and TKO cells during PGCLC differentiation in the presence and absence of cytokines (RNA-seq reads normalised by DESeq2). Data points: biological replicates, centre: mean, error bars: standard deviation, $n = 2$. (**F**) Principal component analysis (PCA) of bulk RNA-seq samples from this study: Wild-type and TKO cells at day 0 (EpiLCs), day 2 and day 6 of differentiation (+/− cytokines) ($n = 2$); and data from (Ishikura et al, 2021): EpiLCs and PGCs from days E9.5, E12.5 and E14.5 ($n = 2$).

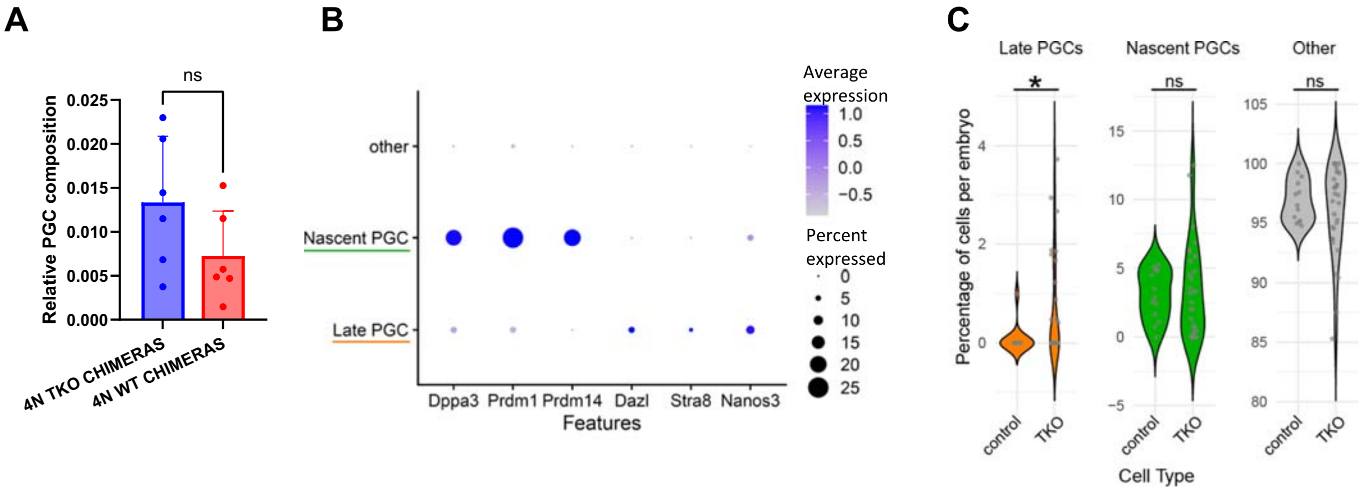

Figure EV6. Single-cell analysis of PGCs in wild-type and TET-TKO chimeric embryos.

(**A**) Relative PGC composition for whole-embryo chimera with transcriptional age >7.75, data from Table S1 in (Cheng et al, 2022). Data points: individual embryos, centre: mean, error bar: standard deviation, $n = 6$. The difference between genotypes was not statistically significant using the Student's *t*-test (ns indicates *p* value >0.05). (**B**) Expression of germline markers in two newly identified cell clusters: "nascent PGCs" and "late PGCs", all other cells within whole-embryo chimeras classified as "other". Clustering was performed using both wild-type and TKO cells. Colour intensity indicates level of expression, circumference size indicates the percentage of cells within the cluster that express each gene. (**C**) Percentage of each cell type per embryo (Nascent PGCs, Late PGCs and Other). The total number of cells were matched between WT and TET-TKO genotypes (4376 cells for both). Data points: individual embryos, number of embryos WT = 13 TKO = 30. Significance calculated by Student's *t*-test, ns: *p* value >0.05, Star (*): *p* value <0.05. Individual *p* values are provided in Table EV1.

 © The Author(s)
